# Traumatic Brain Injury in the Long-COVID Era

**DOI:** 10.1089/neur.2023.0067

**Published:** 2024-01-30

**Authors:** Denes V. Agoston

**Affiliations:** Department of Anatomy, Physiology, and Genetics, School of Medicine, Uniformed Services University, Bethesda, Maryland, USA.

**Keywords:** biological reserve, comorbidities, traumatic brain injury

## Abstract

Major determinants of the biological background or reserve, such as age, biological sex, comorbidities (diabetes, hypertension, obesity, etc.), and medications (e.g., anticoagulants), are known to affect outcome after traumatic brain injury (TBI). With the unparalleled data richness of coronavirus disease 2019 (COVID-19; ∼375,000 and counting!) as well as the chronic form, long-COVID, also called post-acute sequelae SARS-CoV-2 infection (PASC), publications (∼30,000 and counting) covering virtually every aspect of the diseases, pathomechanisms, biomarkers, disease phases, symptomatology, etc., have provided a unique opportunity to better understand and appreciate the holistic nature of diseases, interconnectivity between organ systems, and importance of biological background in modifying disease trajectories and affecting outcomes. Such a holistic approach is badly needed to better understand TBI-induced conditions in their totality. Here, I briefly review what is known about long-COVID/PASC, its underlying—suspected—pathologies, the pathobiological changes induced by TBI, in other words, the TBI endophenotypes, discuss the intersection of long-COVID/PASC and TBI-induced pathobiologies, and how by considering some of the known factors affecting the person's biological background and the inclusion of mechanistic molecular biomarkers can help to improve the clinical management of TBI patients.

## Background and Introduction

Major determinants of the biological background or reserve, such as age,^1–5^ biological sex,^6–8^ pre-existing conditions (diabetes, hypertension, obesity, etc.),^9,10^ and medications (e.g., anticoagulants),^11,12^ are known to affect outcome after traumatic brain injury (TBI).

It feels like distant memory, but SARS-CoV-2 (severe acute respiratory syndrome coronavirus 2) and its more transmissible variants, Omicron, its subvariants, BA.4 and 5, etc., have infected >100 million persons in the United States and killed >1 million (https://covid.cdc.gov/covid-data-tracker/#datatracker-home) over the past 3 years. Worldwide numbers are ∼700 million infected and ∼7 million killed (https://covid19.who.int). In addition to the tragic loss of lives directly attributable to coronavirus disease 2019 (COVID-19), SARS-CoV-2 infection can cause long-lasting or even permanent pathobiological changes.^13^ “Long-COVID” or post-acute sequelae of SARS-CoV-2 infection (PASC) is a multi-symptomatic condition that involves virtually all organ systems.^14–16^ Long-COVID/PASC can adversely affect quality of life and it has become a significant public health challenge.^17–19^ It has been shown that long-COVID/PASC can increase one's predisposition to other disorders (e.g., stroke) and adversely affect recovery from various disorders.^16,20^

TBI has been long recognized as the “silent epidemic,”^21,22^ but given its global nature, it should rather be called a pandemic. However, we still have limited understanding of the biological background, for example, biological sex^23,24^ or comorbidities.^25,26^ Reflecting the drastic decline in personal mobility attributable to quarantines and lockdowns, the incidence of TBI caused by traffic accidents also declined during the COVID-19 pandemic, but assaults and severe injuries increased.^27^ Importantly, outcomes were adversely affected, mortality rates increased, especially in middle-/low-income countries.^28^

The combination of focusing on developing and testing vaccine(s) for SARS-CoV-2 and public healthcare measures, like social distancing, resulted in major disruptions and/or the complete halt of clinical trials,^29–32^ including TBI.^33^ Compensating for the “lost year(s)” in TBI clinical research attributable to COVID-19 pandemic alone has its own challenges—along with new opportunities.^34^ The increased recognition of long-COVID/PASC as a public health issue has added a new dimension to an already complex health condition arguing for a reassessment of our previous approaches to TBI. The new, post-COVID-19 patient landscape, the additional varying individual vulnerability attributable to long-COVID/PASC to outcomes after TBI will require further stratification of patients. This can be accomplished by using an extended panel of molecular biomarkers, utilizing machine learning (ML)^35^ that can guide and optimize individualized therapeutic interventions.

## Long-COVID/PASC

The real prevalence of long-COVID/PASC is currently not well known because of different criteria used in reporting.^16,36^ The latest Centers for Disease Control and Prevention data indicate that ∼15% of American adults have had long-COVID/PASC and 5.8% of Americans currently have it (https://www.cdc.gov/nchs/covid19/pulse/long-covid.htm). Studies have shown that only ∼10–40% of COVID-19 patients recovered completely 60 days after being discharged from hospitals,^37–39^ and the remaining 60–90% of discharged COVID-19 patients have experienced various symptoms that can be attributed only to the infection with SARS-CoV-2.^40^ Although vaccination protects against severe disease, the level of protection against long-COVID/PASC is currently unknown.^41^ Importantly, even asymptomatic persons infected with SARS-CoV-2 can develop long-COVID/PASC of varying severity, further complicating one's ability to assess its true prevalence.^42,43^ The majority (∼90%) of severely ill COVIID-19 patients have reported long-COVID/PASC symptomatology,^44^ but up to 40% of patients after a mild case of COVID-19 infection have also reported long-COVID/PASC-like symptoms.^13,45^

Although some studies indicated that long-COVID/PASC is relatively independent of the severity of COVID-19,^46^ there is a correlation between disease severity and prevalence of long-COVID-PASC. A Swedish study showed that only 1% of mild COVID-19 patients developed long-COVID/PASC, but 32% of severe cases (intensive care unit–treated persons) showed symptoms 1 year post-infection.^47^ Biological sex also plays an important role; severe COVID-19 with unfavorable outcome mainly affects males, whereas long-COVID/PASC disproportionally affects biological females.^47–50^

Long-COVID/PASC can affect multiple organ systems, and the most frequently reported symptoms are fatigue, shortness of breath, nausea, palpitations, joint and chest pain, and gastrointestinal and gynecological problems.^15,40^ Most patients, however, suffer from neurological and -psychiatric symptoms such as “brain fog,” anxiety/depression, memory loss, inattention, disorientation, disturbances of sleep, and headaches.^14,51–57^ These symptoms are similar to a previously described condition, functional neurological disorders (FNDs),^58^ and similar symptoms were observed after SARS and MERS infections.^59^ Patients with chronic fatigue syndrome and fibromyalgia^60^ as well as post-traumatic stress disorder^61^ and “chronic” TBI/persistent post-concussive syndromes also have a similar symptomatology.^62^ It should also be noted that many of these symptoms can be psychogenic, caused by the emotional, mental, and psychological stress attributable to COVID-19-related shutdowns, quarantines, social isolations, etc. Such a lack of specificity and complexity make diagnosing “organic” long-COVID/PASC both important and challenging. A recent study has found evidence that even vaccination without infection can cause FNDs,^63^ but it is unclear whether the cause is organic or “psychogenic.”

## Candidate Pathomechanism(s) of Long-COVID/PASC

The exact pathomechanism of long-COVID/PASC is currently not known. Candidate mechanisms (summarized in Table 1) fell into two categories: direct and indirect.^64–66^

**Table 1.** List of Pathomechanisms Suspected in the Development of Long-COVID/PASC and Their Potential Effect on the Outcome After Various Severities of TBI

***Pathomechanism(s) (selected)***	***Level of evidence in the pathomechanism of long-COVID/PASC***	***Potential influence/effect on outcome after***		
		***miTBI***	***MoTBI***	***sTBI***
Direct				
Direct viral invasion and/or viral reservoirs in the CNS	Low/uncertain^67,68,244,245^	?	?	?
Definition: presence of the virus and/or viral particles in cells of the CNS				
Indirect				
Endothelial damage/dysfunction/(micro)vascular injury	Moderate to high^64,70,75–77,93^	+++	+++	+++
Definition: abnormal *vascular* functions, more reactive endothelial phenotype, injury, blockage of small vessels including capillaries				
Abnormal coagulation	High (after severe COVID-19)^79,246,247^	+++	+++	+++
Definition: disruptions in the body's ability to control blood clotting				
Inflammation	High (after severe COVID-19)^81,89,107,248–250^	+/–	++	+++
Definition: maladaptive immune response				
Abnormal immunometabolism	Moderate^84–87,251^	+/–	++	+++
Definition: changes in intracellular metabolic pathways of immune cells that alter their functionality				

PASC, post-acute sequelae of SARS-CoV-2 infection; TBI, traumatic brain injury; CNS, central nervous system; COVID-19, coronavirus disease 19.

### Direct mechanisms

Direct mechanism includes, viral invasion, proliferation, and effect of viral particles (e.g., viral proteins) on the various cell types of the brain.^67–69^ There is *in vitro* evidence that SARS-CoV-2 can infect cultured human cerebral microvascular endothelial cells.^70^ Viral particles were found in endothelial cells in SARS-CoV-2-infected non-human primates, and SARS-CoV-2 nucleic acids were detected in supporting cells, choroid plexus, and sustentacular cells, but not in neurons.^71^ It is possible that some, likely immunocompromised persons may have a hidden viral reservoir, which can cause reinfection. Recurrence of SARS-CoV-2 positivity after antiviral treatment by Paxlovid or other SARS-CoV-2 antivirals demonstrates that there can be an undetectably low level of a viral reservoir that can cause COVID-19 to rebound.^72^ Based on our current understanding and available evidence, the hidden cerebral viral reservoir likely exists in only a small subset of long-COVID/PASC patients^73^ and may not be responsible for the majority of long-COVID/PASC cases.^74^

### Indirect mechanisms

The indirect mechanism includes endothelial/vascular damage,^70,75–78^ coagulopathy,^79,80^ and abnormal immune functions like altered immunometabolism, chronic inflammation, immune exhaustion, and autoimmunity.^54,81–90^

### Endothelial pathologies

Endothelial damage/dysfunction/(micro)vascular injury has emerged as a leading candidate pathology of long-COVID/PASC.^78,91,92^ The main target of SARS-CoV-2 is the ACE2 receptor bearing endothelial cells in the central nervous system (CNS),^70,71^ and the vascular dysfunction, endothelial damage, and (micro)vascular injury resulting in dysfunction of blood vessels has been one of the hallmarks of systemic SARS-CoV-2 infection and one of the leading candidate pathomechanisms of long-COVID/PASC.^75–77,93–96^ Endothelial cells are key regulators of cell-to-cell adhesion, blood–brain barrier (BBB) formation, and transendothelial transport, including cell migration, coagulation, and inflammation, involving both humoral and cellular pathways.^76^ Endothelial stress caused by SARS-CoV-2 infection can cause long-lasting changes in the cerebral microvasculature in the forms of microthrombosis and altered BBB functions.^97–100^ In summary, endothelial and (micro)vascular abnormalities appear to be the leading pathomechanisms classifying long-COVID/PASC as a new vessel disease.^78^

### Coagulopathies

Endothelial/vascular stress and damage can lead to coagulopathies and persistent hyper- and abnormal coagulation, one of the hallmarks of acute COVID-19,^101^ but studies have also demonstrated the persistence of coagulopathies in long-COVIC/PASC.^102,103^ The presence of microclots with abnormal protein content, including amyloid, combined with an abnormal fibrinolytic system unable to resolve these clots, can result in a hypercoagulable state, manifested in the circulation of microclots.^103,104^ Studies have hypothesized that this hypercoagulable state is caused by a chronic inflammatory process^104,105^ linking vascular abnormalities and the hypercoagulable state to various forms of immune pathologies.^46,105,106^

### Inflammation

The severe form of COVID-19 infection is characterized by a hyperinflammation “cytokine storm,” which can lead to a chronically altered, maladaptive immune response.^81,107^ The cytokine storm, the hallmark of severe COVID-19, causes—additional—organ damage, the release of damage-associated molecular patterns (DAMPs).^108^ HMGB1, hsp70, mitochondrial DNA, and other DAMPs further activate the inflammatory process, leading to a vicious cycle that, if not completely broken, can exist for months after the acute phase and lead to long-COVID/PASC.^109,110^ Indeed, an altered immune system, characterized by chronically elevated levels of proinflammatory molecules, has been identified as one of the leading molecular pathologies of long-COVID/PASC.^111,112^ Circulating proinflammatory molecules can cause—additional—vascular stress and endothelial activation, leading to fibrinogen accumulation around the vessels, attracting microglia and/or macrophages and inducing a vicious cycle of neuroinflammation and tissue damage in the CNS.^113,114^ Another form of immune pathologies suspected in the pathology of long-COVID/PASC is altered immunometabolism.^86^ SARS-CoV-2 infection can modify any of the six major metabolic processes that compromise their functionality, leading/contributing to a chronic inflammatory stage.^84,85,87^

### Autoimmunity

#### Autoimmunity in long-COVID/PASC

Immunedysregu-lation, especially autoimmunity, has been increasingly recognized as one or the main pathologies responsible for long-COVID/PASC.^83,115,116^ Several studies have indicated that SAR- CoV-2 infection can result in an unbalance of the affected person's immune homeostasis, resulting in the development of autoantibodies potentially leading to the development autoimmune diseases.^116–118^ Autoantibodies against brain-tissue–specific epitopes have been found in long-COVID/PASC patients, and their presence correlates with neuropsychiatric abnormalities.^119^ During the acute phase of SARS-CoV-2 infection, serum levels of neural injury markers of GFAP and NF-L were elevated and, importantly, remained elevated in patients who could be classified as suffering from long-COVID/PASC.^120–122^ Elevated levels of neural injury markers were associated with elevated levels of inflammatory cytokines and IgM autoantibodies against, for example, myelin-associated glycoprotein and many other brain-specific proteins.^119,123^ Abnormal protein homeostasis, combined with protease activation and hyperinflammation, can significantly contribute to the autoimmune mechanism,^124,125^ the generation of autoantibodies against brain-specific proteins.^16,115,126^

In summary, current evidence points toward three major inter-related pathomechanisms (vascular/endothelial stress/damage, abnormal coagulation, and inflammation) that are the most likely underlying pathologies of long-COVID/PASC. Some of these pathomechanisms may occur individually, but they can also overlap, and can also change over time.

## TBI: A Spectrum of Disorders of Different Disease Endophenotypes

TBI is not a single disease, but a spectrum of disorders with the same causation: physical insult to the head and brain.^127–132^ The only similarity between an unconscious patient with skull fracture, subdural hematoma, and brain contusion and a patient walking into an emergency room with a bump on his or her head feeling dizzy is that there was a physical impact—of different intensities and kinds—to the head.^133^ Thus, there are two dimensions of TBI that need to be considered; one is severity, traditionally addressed by the Glasgow Coma Scale introduced in 1974 by Teasdale and Jennett.^134^ However, our biological understanding of the pathophysiological mechanisms underlying the functional abnormalities have become substantially more refined since 1974.^135–138^ Mild TBI, clinically also called concussion, only causes temporary perturbance of cellular structures and may dislocate membrane-bound ion channels, receptors, and/or intracellular organelles, causing typically transient molecular disturbances reflected in metabolic abnormalities that clinically manifest as a temporary altered state of consciousness.^139,140^ After moderate TBI, biomechanical forces cause substantial direct tissue and cell damage and cell death, majorly disrupting neuronal signaling and networks manifesting clinically as prolonged loss of consciousness.^141^ Severe TBI, frequently comorbid with polytrauma, causes major loss of brain parenchyma, severe disruption of neuronal networks, loss of consciousness, and severe neurological dysfunctionality.^142–145^

The second critical dimension is the temporal aspect of TBI-induced pathobiological changes.^146^ Though the exact temporal profile of these changes is currently not well understood, available data indicate a dynamic and complex pattern of TBI-induced pathobiological changes that can span weeks or months.^147–153^

## Major Pathobiologies Triggered by TBI: The Disease Endophenotypes

The physical impact to the head—depending on the intensity and type of injury—triggers a variety of pathobiological changes that can occur in partly overlapping fashion, interact in a highly complex fashion, and change over time.^154,155^ Penetrating TBI causes massive tissue damage, cellular death, and bleeding that release intracellular molecules called damage-associated molecules (DAMPs). DAMPs, like HMGB1, mitochondrial DNA, and S1008/9, rapidly activate the innate immune system by Toll-like receptors (TLRs) with the aim to remove tissue debris and restore homeostasis.^156,157^ However, the inflammatory process can continue beyond the acute phase and transform into a chronic process.^158^

Axonal injury is a hallmark of diffuse TBI, but the extent of damage varies from major white matter loss clinically manifested in severe functional deficits to temporary, molecular-level disruption of axonal structures, causing mild and transient neurobehavioral abnormalities.^159–162^

An important but currently poorly understood TBI (endo)phenotype is characterized by injury to the cerebral vasculature.^163–166^ The effect of diffuse TBI on the vasculature can range from endothelial stress, damaged BBB function, microbleeding, and hemorrhage.^167–169^ These pathobiological changes have been detected by neuroimaging^163,170^ and also by elevated plasma levels of protein biomarkers of endothelial and vascular stress and damage (VEGF, vWF, and cFN)^163,166,171–175^ and/or endothelial tight junction proteins (Claudin-5 and Occludin).^150,176,177^ These TBI-induced vascular changes are important inducers of downstream mechanisms such as activation of blood clotting and the innate immune system.^178–183^ Inflammation is a key adaptive response to any kind of noxious stimuli in all multi-cellular organisms,^184,185^ and the neuroinflammatory response to TBI—including mild, especially repeated mTBI—is emerging as a key pathobiology responsible for adverse outcomes.^183,186,187^ The neuroinflammatory response to TBI includes both humoral and cellular players.^183^ Depending on the type (e.g., closed, diffuse, or penetrating) and severity of the injury, the cellular components can include only intracranial cellular population, microglia, and astroglia or, after penetrating injury, peripheral immune cells (e.g., macrophages also contribute to the inflammatory response).^188^

Astrocytes play an especially important and complex role in the neuroinflammatory process; they are involved in regulating both innate and adaptive immune responses after TBI.^189–191^ In the activation of astrocytes, astrogliosis subsequent to penetrating TBI demarcates the injury site and a highly complex bidirectional signaling process between astrocytes and microglia is a key regulator of the acute and chronic neuroinflammatory response.^189–191^ Moderate and severe TBI disrupt—to varying degrees—the BBB causing the exposure of brain-specific molecules to the adaptive immune system, generating potentially detrimental long-lasting cellular and humoral responses that can cause or contribute to chronic adverse conditions.^183,192,193^

## Intersection of Long-COVID/PASC and TBI-Induced Pathobiologies

Several of the suspected pathobiologies underlying long-COVID/PASC have the potential to affect recovery after various forms of TBI (Table 1). A major challenge for the clinical management of TBI patients has long been the heterogeneity of patients differing, for example, in age, biological sex, comorbidities, comedications, and cofactors—such as alcohol and/or drugs—and pre-existing conditions, such as long-COVID/PASC. The outcome after SARS-CoV-2 infection, especially after the severe form, has shown a sharp age dependence; elderly persons have shown significantly poorer outcome,^194,195^ but older age as a risk factor for developing long-COVID/PASC has been questioned.^196^ Conversely, young age has been shown as “protective” against the severe form of COVID-19,^194^ but not against developing long-COVID/PASC.^197,198^ Pediatric and adolescent populations represent a significant percentage of TBI cases,^199^ and it is known that TBI adversely affects later phases of neuronal development.^4,200–203^ The question is of what unknown is the effect of SARS-CoV-2 infection and/or long-COVID/PASC on the outcome of TBI, specifically the mild/concussive form that is the most frequent among adolescents/young adults. Elderly persons, representing the other predominant age group in TBI, has especially poor recovery after TBI, as reported by the large TRACK-TBI study,^8^ and long-COVID/PASC can further diminish the recovery process.

Biological sex seems to play a significant role in outcome after SARS-CoV-2 infections. Severe COVID-19 with an unfavorable outcome was observed mainly in males, whereas long-COVID/PASC disproportionally affected biological females.^47–50^ The large TRACK-TBI study has found that women are more vulnerable to develop persistent neurobehavioral symptoms than men after mTBI and suffer from chronic post-concussion syndromes.^8^ Accordingly, women suffering from long-COVID/PASC will likely have poorer long-term functional outcomes after TBI.

There are currently very limited data on how long the long-COVID/PASC really lasts and whether it has distinct disease phases.^45^ The current view is that long-COVID/PASC is a chronic condition with relatively steady pathobiologies,^15,103^ but there are reports indicating time-dependent changes in the underlying pathobiologies.^44^ TBI induces severity- and injury-type–dependent pathobiological responses that change dynamically over time, although the exact temporal pattern of these changes is currently poorly understood.^153,182,193,204^ Accordingly, not all the pathologies are present at every post-injury period,^148,150–152^ and their co-occurrence with pathologies of long-COVID/PASC—especially an abnormal inflammatory profile—has the potential to negatively affect the recovery process. SARS-CoV-2 infection itself can cause brain injury—defined as elevated serum levels of brain-injury markers NF-L, Tau, and GFAP—during the acute phase of COVID-19.^122^

Elevated levels of these brain injury biomarkers were associated with elevated serum levels of inflammatory cytokines (TNFa, IL-1b, and IL-6) and autoantibodies—both IgM and IgG—against brain-specific proteins (e.g., myelin-associated glycoprotein). Importantly, 4 months after the infection, the convalescent phase that can qualify for long-COVID/PASC, serum levels of brain injury markers, especially Tau, were still significantly elevated over normal controls along with inflammatory cytokines and autoantibodies. These findings implicate an ongoing inflammatory and autoimmune process, given that manifestations of immune dysregulation, one of the main pathomechanisms of SARS-CoV-2 infection, are also suspected in causing long-COVID/PASC.^83,118,205^

**FIG. 1.**
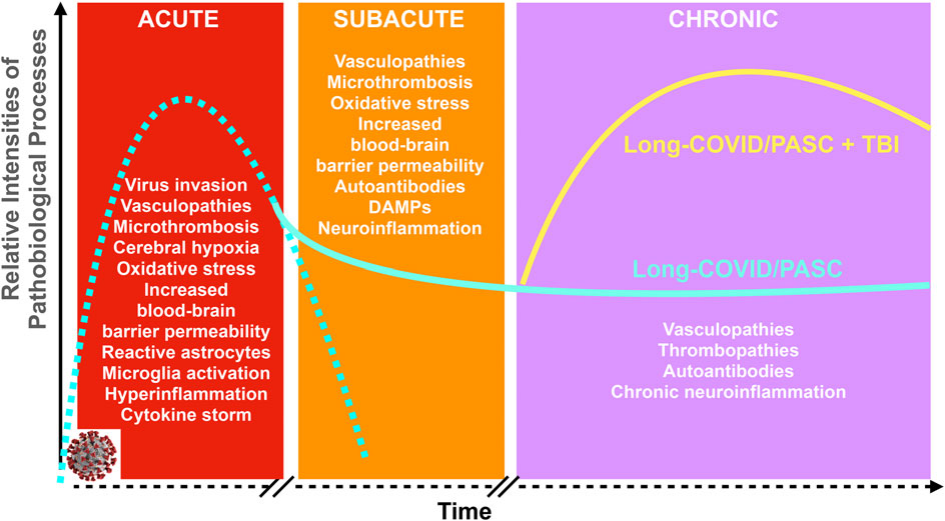
Onset and extent of major pathobiological changes during the various phases of severe COVID-19 and the hypothesized role of long-COVID/PASC affecting the outcome of TBI. The pathobiological changes identified or suspected during the acute, subacute, and chronic (long-COVID/PASC) phases of COVID-19 are listed. The dashed turquoise line indicates the relative intensity and relative timeline of pathobiologies of COVID-19. The solid turquoise line indicates the relative intensities of pathobiological changes associated with long-COVID/PASC. The solid yellow curve indicates the relative intensity and temporal changes of pathobiologies of TBI on a long-COVID/PASC background. Relevant references are in the text and listed in the references. DAMPs, damage-associated molecular patterns; PASC, post-acute sequelae SARS-CoV-2 infection; TBI, traumatic brain injury.

The altered biological background in long-COVID/PASC characterized by vascular abnormalities and, importantly, by a chronic inflammatory landscape can majorly affect the disease process after TBI (Fig. 1). An ongoing neuroinflammation has been proposed as the key pathomechanism of chronic TBI and TBI-induced pathological processes (e.g., chronic traumatic encephalopathy and Alzheimer's disease).^191,206^ Additional parenchymal damage caused by TBI will take place on an already dysregulated immune system causing additional and/or maintaining the long-term parenchymal damage, releasing DAMPs, further activating the adaptive immune response through TLR signaling and resulting in a vicious cycle that delays and or adversely affects the recovery process after TBI.^120,122^

## The Role of Biomarkers

Identification of TBI endophenotypes is a critical step toward developing evidence-based individualized clinical management of TBI patients.^166,207–209^ Because of their rich information content, blood-based (and other biofluid) protein biomarkers have currently the most potential to perform molecular phenotyping of TBI patients.^210–214^ In order to identify increased vulnerability of TBI patients attributable to pathologies underlying/suspected in long-COVID/PASC, an expanded biomarker panel should be used. Such a panel should include markers of any of the pathomechanisms suspected in the development of long-COVID/PASC (Table 2). These markers should be in addition and used in the context of the current, most commonly used, well-established markers of neural injury (GFAP, UCH-L1, Tau, and NF-L).^211^ There is an increasing recognition to use “mechanistic” biomarkers in TBI in conjunction with “classical” injury markers.^215^ However, markers of coagulopathies (e.g., vWF, D-dimer), endothelial stress (e.g., VEGF-A, Ang-1/2, and ET-1), vascular damage (CLDN5, Occludin, VCAM-1, and cFN),^166,174,216—220^ and inflammation (e.g., CRF, IL-1b, IL-6, TNFa, IL-8, and CXCL12)^84,221—223^ that have already been used in clinical settings should be coanalyzed with the panel of injury markers. The caveat is that elevated serum levels of the neural injury marker NF-L have been found in patients who could qualify as suffering from long-COVID/PASC (4 months after infection). This finding indicates an ongoing neuronal damage likely caused by an ongoing inflammation.^120–122^

**Table 2.** Biomarkers of Selected Pathomechanisms Suspected in PASC for Blood-Borne Phenotyping of TBI Patients

***Biomarker of***	***Full name, abbreviation, references, and notes***
Autoimmunity	Cyclic citrullinated peptide (CCP),^252^ protein microarray-based assays^253,254^
Abnormal immune response; altered immunometabolism, immune dysregulation, hyperinflammation	Chemerin (ChM)^84,221,222^
	C-reactive peptide (CRP), tumor necrosis factor alpha (TNF-a), interleukin-1 beta (IL-1b), interleukin-6 (IL-6)^223^
Endothelial (vascular) damage, abnormal coagulation	von Willebrand factor (vWF), IL-18, vascular endothelial growth factor (VEGF), cellular fibronectin (cFN), D-dimer (Dd), fibrinogen (Fb)^166,174,216–220^

PASC, post-acute sequelae of SARS-CoV-2 infection; TBI, traumatic brain injury.

The major challenge will be how to analyze, harmonize, and correlate the large volume of—various—biomarker data in order to help clinical decision making. Potential solutions should include massive use of Big Data approaches like ML.^35,207,224^ Critically, any successful ML approach critically relies on a high quality and high quantity of primary-protein, physiology, imaging, etc., biomarker data and well-structured/machine-readable clinical reports.^35,225–227^

The critical unmet need is the availability of normal, reference ranges of biofluid blood—plasma and serum—and cerebrospinal fluid–based protein biomarkers. Astonishingly, no such publicly available database exists. Combined with issues of pre-analytical and analytical variables (e.g., different assay platforms),^228^ the current use of biomarker data has limited value.

## The Role of TBI Models

Pioneering works have developed high-fidelity animal models of various forms of TBI,^229–234^ enabling one to identify the anatomical, cellular, and molecular substrates of the primary and secondary injury processes.^185,235–237^ These experiments have—mostly if not exclusively—been performed using healthy young male rats and only recently females have also been included.^238,239^ Though studies have started to address how the young, developing brain responds to and recovers from TBI,^240^ much less is known about the aging brain's response^241^ despite the huge and increasing number of aging persons affected by TBI.^242^ These elderly patients frequently suffer from comorbidities (e.g., long-COVID/PASC) and under (multiple) medications that combined can substantially alter patients' biological background in addition to age.^26,243^ In order to generate clinically relevant, translatable information, there is a need to develop and use animal models of human conditions/diseases (e.g., chronic inflammation) that are prevalent in the aged population.

## Summary

Long-COVID/PASC has been identified as a significant public health issue. It reduces the quality of life, increases the vulnerability of affected persons to other diseases, and negatively affects disease processes. The underlying—suspected—pathologies, vascular abnormalities, coagulopathies, and inflammation can adversely affect the recovery process after TBI. Expanded diagnostics aimed to better inform about the biological background and comorbidities, such as long-COVID/PASC, of TBI patients will help to develop individualized clinical management.

## Abbreviations Used

BBB: blood–brain barrier
CNS: central nervous system
COVID-19: coronavirus disease 2019
DAMPs: damage-associated molecular patterns
FNDs: functional neurological disorders
ML: machine learning
PASC: post-acute sequelae SARS-CoV-2 infection
SARS-CoV-2: severe acute respiratory syndrome coronavirus 2
TBI: traumatic brain injury
TLRs: Toll-like receptors
